# Maltitol: Analytical Determination Methods, Applications in the Food Industry, Metabolism and Health Impacts

**DOI:** 10.3390/ijerph17145227

**Published:** 2020-07-20

**Authors:** Ariana Saraiva, Conrado Carrascosa, Dele Raheem, Fernando Ramos, António Raposo

**Affiliations:** 1 Department of Animal Pathology and Production, Bromatology and Food Technology, Faculty of Veterinary, Universidad de Las Palmas de Gran Canaria, Trasmontaña s/n, 35413 Arucas, Spain; 2 Northern Institute for Environmental and Minority Law (NIEM), Arctic Centre, University of Lapland, 96101 Rovaniemi, Lapland, Finland; 3 Pharmacy Faculty, University of Coimbra, Azinhaga de Santa Comba, 3000-548 Coimbra, Portugal; 4 REQUIMTE/LAQV, University of Oporto, 4051-401 Porto, Portugal; 5 Department for Management of Science and Technology Development, Ton Duc Thang University, Ho Chi Minh City, Vietnam; 6 Faculty of Environment and Labour Safety, Ton Duc Thang University, Ho Chi Minh City, Vietnam

**Keywords:** food additives, food industry, food safety, health impacts, maltitol, metabolism, sweeteners

## Abstract

Bulk sweetener maltitol belongs to the polyols family and there have been several dietary applications in the past few years, during which the food industry has used it in many food products: bakery and dairy products, chocolate, sweets. This review paper addresses and discusses in detail the most relevant aspects concerning the analytical methods employed to determine maltitol’s food safety and industry applications, its metabolism and its impacts on human health. According to our main research outcome, we can assume that maltitol at lower doses poses little risk to humans and is a good alternative to using sucrose. However, it causes diarrhoea and foetus complications at high doses. Regarding its determination, high-performance liquid chromatography proved the primary method in various food matrices. The future role of maltitol in the food industry is likely to become more relevant as processors seek alternative sweeteners in product formulation without compromising health.

## 1. Introduction

Maltitol (C_12_H_24_O_11_; 4-O-α-glucopyranosyl-D-sorbitol) is a hygroscopic non-reducing sugar and disaccharide polyol that is listed as an alternative sweetener to sugar because, except for browning, it possesses roughly 75–90% of sucrose’s sweetness and has similar properties [1]. Of all polyols, maltitol has the closest solubility curve to that of sucrose and is freely soluble in water: 220 g of sucrose is soluble in 100 mL of water at 37 °C, whereas 200 g of maltitol is soluble in 100 mL of water at 37 °C. Once dissolved, the viscosities of sugar solutions and maltitol are equivalents, with viscosities of 18 millipascal seconds (mPa.s; 50% solution in water at 20 °C) and 23 mPa.s, respectively. Comparable solubility helps maltitol to dissolve in the mouth in almost exactly the same way as sucrose, leaving the mouth able to feel the expected sweetened taste of a given food product [2].

Given its high crystalline purity and chemical composition, in its natural crystalline form maltitol is less hygroscopic than sugar. At about 40 °C, maltitol absorbs ambient moisture even at a relative humidity of 82% and higher, as opposed to 80% for sucrose. This would mean improved shelf stability of those goods made with maltitol rather than sucrose when processed under given atmospheric/climate conditions. When employed as a covering on confectionery and chewing gum, maltitol’s low hygroscopicity leads to long-lasting crunchiness. Reduction in the carbonyl group enhances maltitol’s thermo-chemical stability during the conversion from maltose into maltitol. It does not react with amino acids when heated, which avoids Maillard reactions and, thus, lowers excessive browning potential [2].

Maltitol occurs naturally in different fruits and vegetables. Small amounts of maltitol naturally exist in roasted malt and chicory leaves. Maltitol is commercially produced from the starch of cereals such as corn, wheat and potatoes. Manufacturers resort to D-maltose catalytic hydrogenation to create hydrogenated disaccharide composed of a glucose molecule and a sorbitol molecule that are bonded together [2,3].

As with other sugar alcohols, maltitol is poorly absorbed in the small intestine, and has lower insulinaemic (35 vs. 45) and glycaemic indices (35 vs. 68), and a lower caloric value (2.4 vs. 4 kcal/g) and sweetening power (approx. 90%) than sucrose [4,5]. The metabolism of maltitol follows a known pathway [6,7,8]. This compound is partly absorbed only in the proximal intestine and enters the lower intestine and colon. As a result, digestive tolerance to maltitol has been previously examined in chocolate in healthy adult volunteers [9,10,11]. Adults can eat as much as 40 g of maltitol/day with no significant symptoms, while children can consume 15 g [9,10,11,12]. So maltitol is used primarily as a sugar substitute in food products as it has a bulking effect compared to intense sweeteners [3]. Maltitol is also employed in pharmaceuticals or oral care products (toothpaste) [13,14]. Apart from its technological and nutritional qualities, maltitol also possesses similar organoleptic properties to glucose [15] and provides good digestive tolerance. This permits its widespread use for both children and adults in various dietary applications, mostly in the sweet food categories such as cakes, pastries, sugar confectionery, chocolate, chewing gum and snack bars as well as its use as a tabletop sweetener [10,12,16]. Maltitol exhibits certain prebiotic effects in rats or humans [17,18]. As nine hydroxyl groups exist in the molecule, it is reasonable to believe that maltitol is able to act as an additive to avoid moisture loss and to further delay stalling in foods like bread [1].

As very little work on maltitol can be found in the literature, this paper aims to review analytical methods for its determination, its chief food industry and safety applications, and its metabolism and impacts on human health that stem from its utilisation.

## 2. Analytical Methods for Maltitol Determination

Assessing maltitol in food and beverages with low/no sugar content is relevant in both nutritional and quality control terms. The analytical procedures followed in sugar alcohol analyses are similar to those employed for other sugars. Nonetheless, sugar alcohols are characterised by high chemico-thermal stability (up to 180 °C). High-performance liquid chromatography (HPLC) methods are the most widespread choice thanks to their robustness, high sensitivity and easy sample preparation [19]. However, maltitol entails several analytical problems, as we discuss below.

Food products are complex matrices given major differences in their composition, which comprises several types of thickeners, preservatives, macromolecules, and colour additives. As many food matrix components have similar polarities to maltitol, this compound is not easy to isolate. Moreover, the maltitol levels encountered in some food products entail adjusting the sample concentration to the analytical method’s linear range by dilution [19].

An analytical method for determining a target compound in a given matrix usually goes through three main stages: (1) sampling; (2) sample preparation; and (3) determination of the analyte. In the first stage, it is essential to ensure that the samples are representative, taken without contamination and transported swiftly and properly to the laboratory. When samples are not analysed immediately, storage conditions must be established to avoid changes in the sample quality. The second stage, sample preparation, comprises the process of isolating the compound of interest from interferents in the matrix prior to analysis, with the most suitable instrumental tools. This step is critical for the determination of the analyte, in this case maltitol, and is usually the most time-consuming process to conduct and optimise [19,20]. The sample preparation method depends on the complexity of the matrix, but in general homogenisation, extraction, clean-up and pre-concentration may be required, depending on sample complexity, to remove chemical interferences and determine whether or not a sample contains maltitol and at what concentration [21,22].

Preparing liquid samples is normally simple, but this is limited to dilution and filtration. In already filtered samples, injection is frequently performed directly. Carbonated drinks need to be degassed in an ultrasonic bath, either under vacuum or by sparging with nitrogen [19,23]. Some sample solutions may need to be treated with clarifying agents, such as Carrez solutions, which are suitable for the elimination of suspended solid material, proteins and lipids. Organic solvents, such as ethanol and acetonitrile, are used to selectively precipitate thickeners and polysaccharides. Besides, during sample preparation, acids such as acetic, metaphosphoric and formic acid can also be employed to precipitate food matrix components or to adjust the pH of the sample [20,22]. Whatever the procedure, filtration is always mandatory before the final analysis [21]. A single filtration step with a membrane filter is often enough to achieve the desired characteristics, but sometimes preliminary filtration using filter paper or centrifuge may be necessary [24]. All samples must be subjected to replicated analysis.

Grembecka et al. performed analysis of four sugars and five sugar alcohols, including maltitol, in fruit juices, fruit drinks, nectars and syrups [25]. The sample pretreatment applied was the slightest; it consisted of a first filtration through filter paper to remove particulate matter, followed by a dilution with 75% acetonitrile, and finally a second filtration with 0.45 µm membrane filters [25]. This minimal sample preparation procedure is suitable for many simpler samples and has the advantage of being economical, simple and speedy.

The addition of an extraction step is the most widely used sample preparation technique. For this purpose, the most commonly employed organic solvents are methanol, acetonitrile and chloroform [20]. Liquid-liquid extraction (LLE) can be employed because it is inexpensive but is not easy to automate and normally requires large quantities of solvents. More complex solid matrices may require more elaborate extraction and purification techniques such as solid phase extraction (SPE) or ultrasound-assisted extraction (UAE) with different solvents, even though extraction with water suffices in some cases [19,23].

Andersen et al. analysed four sugars and six sugar alcohols, including maltitol, in several types of matrices, namely desserts, cakes, candies, liquorice, wine, gums, chocolate and pastilles [26]. The samples were ground and the extraction was carried out with water at 60 °C for 4 h at room temperature, followed by centrifugation and filtration through a folded filter (S&S, 592.5, diameter = 125 mm), dilution and a second filtration through a Minisart 0.2 µm [26]. As can be seen from this case, heat is sometimes applied to aid in the extraction process. Overall, the adopted sample preparation protocol was relatively simple.

A quite different approach was used by Nojiri et al., which reported the analysis of five sugar alcohols, including maltitol, in confectionery products [27]. In this case, sample preparation consisted of extraction with 30% ethanol, followed by centrifugation, evaporation, and derivatisation with a 10% solution of p-nitro-benzoyl chloride. The reagent excess was eliminated and the sample was evaporated. Finally, the residue was dissolved in chloroform and purified in an SPE (solid phase extraction) cartridge (Sep-Pak C18) [27]. The use of ethanol is advantageous in relation to water, as it inhibits metabolic enzymes, thus contributing to the preservation of the sugar composition [23]. SPE is a well-established tool for the pre-concentration and clean-up of target compounds from aqueous samples and extracts. The bases of this technique are identical to those of conventional liquid-solid chromatography (LSC) and HPLC [22]. The most highly employed SPE cartridges for the extraction of sweeteners from foods are those with columns packed with octadecylsilyl silica (ODS-C18) [21,24]. Briefly, the SPE protocol consists in conditioning the cartridge, followed by the loading of the extract, and finally the washing of the cartridge. Ultimately, the elution of the compounds of interest is carried out with a suitable solvent. It is crucial that interfering compounds are not trapped and elute almost immediately, or that they have a high affinity for the stationary phase in order to be strongly adsorbed. It is worth mentioning that the sensitivity of the final analysis can be increased through a pre-concentration strategy, that is, by evaporation of the eluate until dryness and re-dissolving it in a small volume of solvent suitable for the subsequent analysis [20,21,24]. The solubility of maltitol in the processing solvent is naturally of major relevance.

In general, SPE sample preparation procedures are a good option for samples of a more complex nature (“dirty samples”), as they are undemanding, reasonably inexpensive and swift [21,24]. Moreover, owing to the lower column sizes and volumes, they require the use of less mobile phase volume, which leads to both less waste and less exposure of laboratory technicians to organic solvents, as well as leading to additional cost savings. The sample capacity and eluant volume are usually suitable also for direct injection into HPLC equipment without the need for additional sample preparation processes, thereby reducing both the risk of sample loss and contamination [22]. Furthermore, SPE exhibit greater potential for selective isolation, i.e., sample fractionation into different compounds or classes of compounds [28]. Finally, SPE systems have been increasingly improved in terms of throughput, precision and accuracy, and are compatible with the most commonly used analytical instrumentation [21].

In another study, Joshi et al. developed a method for the quantification of maltitol in flavoured milk, burfi and yoghurts [29]. Sample extraction was carried out with water under sonication for 20 min at 40 °C. A treatment with Carrez reagents was applied to remove proteins, followed by filtration through filter paper and a 0.22 µm syringe filter [29].

UAE is an efficient, economical and green technique for sample preparation. When compared to the classic extraction procedures, it allows the reduction of the amount of solvent and glassware used, as well as of the extraction time, wherefore it has been increasingly used. This technique explores the cavitation process that is observed when an extractive solvent in contact with a solid matrix is exposed to ultrasound energy. The mechanical effect generated induces a greater solvent penetration into the solid, and causes its mechanical erosion, which results in a superior mass transfer, with a consequent increase in the efficiency of the sample’s extraction and a good recovery of the analyte [30].

After the sample extraction process, certain components of the food matrix may still be contained in the extract and act as a source of interference in the final determination. These undesirable compounds can be co-extracted with maltitol owing to their similar solubility profile. The occurrence of chemical interferents in the extract may bring about accuracy problems in the method, wherefore additional clean-up steps may be essential to obtain proper separation, detection and quantification [21]. As seen in the examples above, precipitation is a commonly applied technique, usually with Carrez solutions, sodium hydroxide/zinc sulphate and similar agents, followed by filtration or centrifugation. Other common clean-up methods include SPE, as earlier discussed, LLE and dialysis. An adequate extraction/clean-up process improves the recovery of maltitol and is indispensable to avoid problems such as clogging of the HPLC columns [21].

To summarise, proper sample preparation/clean-up can shorten analysis time, improve method sensitivity and selectivity, and optimise maltitol identification/quantification. The addition of a pre-concentration step can be interesting, as it can lower the detection limits during the analysis. On the other hand, a filtration step before the final analysis, usually with 0.45 µm filters, is essential for all samples, in order to remove particulate matter that can cause damage to the equipment and interfere with the results [20]. It is recommended also that a guard column filled with the same material as the main column be used, in order to act as a filter, prolong the lifetime of the analytical column and improve its performance [31]. Besides, ultrapure solvents should preferably be used in sample preparation, as this provides greater purity, stability and durability of the analytical instruments [20]. Lastly, the sample preparation procedure chosen to determine maltitol depends on the nature of the matrix and the analytical system to be used in quantification and can be more or less costly and demanding. As general rule, it should be kept as simple as possible to reduce error.

As a final remark, it should be noted that, even though the traditional sample pretreatment methods are still widely used, there is a growing trend in this field to minimise the use of organic solvents and sample sizes, and adopt extraction procedures that allow the analysis of compounds of different classes simultaneously, ideally likely to be automated [20].

That said, and once the correct sample preparation is complete, the foodstuff is ready for analysis. Research papers have reported different approaches to determine maltitol in food products, as discussed below. Table 1 summarises details of practical applications.

Assessing maltitol by ultraviolet (UV) detectors is challenging because its structure lacks chromophore groups. However, UV detectors are not expensive and this obstacle can be overcome by the derivatisation technique using a chromophoric reagent. Nojiri et al. developed a method to analyse six sugar alcohols, including maltitol, in several confectionery products by HPLC after nitro-benzoylation [27]. Using p-nitro-benzoyl chloride permitted maltitol to be converted into a strong UV absorbing derivative [27]. Despite these good results, having to apply derivatisation is a disadvantage that should be considered for routine analyses. This technique increases sensitivity and selectivity, but is lengthy, and the presence of reagents in samples can negatively impact on analysis results. Many foods contain UV active compounds and can cause interference [36].

Another detector that has been described for maltitol determination is the refractive index (RI). The RI detector allows simple and fast analysis of compounds that change the refractive index of the solvent, which are virtually all analytes [34]. Joshi et al. successfully established a procedure for maltitol analysis in dairy products, which was based on the use of an amino column containing a silica-based aminopropyl bonded stationary phase linked with an RI detector [29]. An isolation method based on enzymatic treatment with β-galactosidase was applied for the lactose hydrolysis, which allowed the removal of the lactose peak that had a retention time coincident with maltitol [29]. Hadjikinova et al. also developed and validated an HPLC-RI method for the simultaneous determination of four sugars and five sugar alcohols including maltitol in desserts [32]. Briefly, the sample preparation consisted of extraction with distilled water (50 °C) in a water bath at 60 °C for 15 min, precipitation with sodium hydroxide and zinc sulphate, and filtration (0.45 µm membrane filters). In this case, a column specially designed for the separation of sugars and sugar alcohols (Shodex Sugars SP0810) was employed [32].

The main problem of RI detection is its limitation to isocratic methods. An RI detector is very sensitive to changes in temperature and flow rate [23,34]. Ultraviolet–visible (UV/VIS) detection with a diode array detector (DAD) presents similar limits of detection (LODs) to those of the HPLC-RI procedure, but implies pre- or post-column derivatisation. Hence a gradient elution can be employed, but sample preparation is more time-consuming [34]. Another option is HPLC coupled with fluorescence detectors (FDs), which yields higher selectivity and sensitivity than those of HPLC-DAD, but FDs are costly and require derivatisation [34].

Evaporative light scattering detectors (ELSD) also have been employed. The mobile phase in ELSD is nebulised by air or nitrogen to produce particles, and the light scattered by the resulting particles is measured [23]. Koh et al. developed a method with both an amide-based column and ELS detection [33]. Sample extraction was performed with water at 80 °C for 30 min for gums and candies, and with 50% alcohols at 80 °C for 30 min, after fat removal, for chocolate and processed chocolate products, followed by centrifugation and filtration (0.22 µm PVDF syringe filter) [33]. Unlike amino-based columns, amide-based columns are able to retain analytes over a wide pH range in the mobile phase [38]. These authors tested columns of various lengths (50, 100, 150 mm) and recommended employing the longest column because shorter lengths negatively impact resolution [33]. Nonetheless, the response peaks achieved in the longer columns are often wider, which means less sensitivity given increased diffusion [38]. This method resulted in separation of eight sugar alcohols and five sugars within 15 min without derivatisation, which is a noteworthy outcome. Ethanolamine and triethylamine were added to eluents to modify the stationary phase. The developed analytical system was applied to commercial samples of gums, chocolate, sweets, processed snacks and chocolate products at 0.21–46.41% of sugars and sugar alcohols. Maltitol appeared in gums and sweets [33].

Compared to the RI, ELSD offers much a higher sensitivity and stability of the chromatographic baseline, even in the gradient eluent mode. The required sample pretreatment is normally minimal [23]. Having said that, the pulsed amperometric detector (PAD) and the Corona charged aerosol detector (CAD) are considered superior to ELSD and RI detectors in sensitivity, selectivity and reproducibility terms [25,31].

CAD has the following characteristics regardless of the chemical properties of the compound of interest: high sensitivity to mass, gradient compatibility, wide dynamic range, high precision, and consistent response [23]. CAD works on the principle of the eluent’s nebulisation with nitrogen to produce analyte particles. This is followed by drying to eliminate the mobile phase. Particles are then charged with a high-voltage corona wire. The quantity of charge measured by an electrometer is proportional to the quantity of the analyte of interest in the sample [23,34].

CAD mobile phases must be volatile as they are for ELSD [23]. Grembecka et al. successfully and simultaneously determined by the HPLC-CAD method five sugar alcohols and four sugars, including maltitol, in drinks and dietary supplements, with no extraction step [25]. After analysing commercial samples, maltitol was found only in syrup (dietary supplement) [25].

The hydrophilic interaction liquid chromatography (HILIC) mode is an alternative to HPLC for separating polar compounds like maltitol. The principle of separation is in accordance with the differential distribution of the compounds between a relative hydrophobic eluent and a water rich layer immobilised in a hydrophilic stationary phase [39]. More recently, Pitsch et al. set up a HILIC-CAD method that allows for the simultaneous determination of 30 compounds. It comprises five ions, 17 sugars and seven sugar alcohols, including maltitol, in 24 food and beverage samples [34]. The applied pretreatment was a minimal dilution in 60% acetonitrile with centrifugation. Samples with gas were degassed in an ultrasonic bath prior to dilution. Analyte separation was done in an amide-based column. The use of 60% acetonitrile led to some matrix interference in complex beer samples without affecting the quantification results. It has been suggested that matrix interferences could be further decreased by employing SPE or increasing the acetonitrile concentration in the sample diluent. Separating maltitol from sugars and other sugar alcohols is quite challenging owing to chemical similarities and implies that high-efficiency separation columns and long runs are normally necessary. The above-cited authors took a quantification approach based on peak height instead of peak area, which was successful. They obtained results during a shorter period and did not compromise reliability. The method was generally a sound tool for routine analyses [34].

Sugar alcohols, including maltitol (pKa 12.84), possess weak acid properties, with ionising under high pH conditions, at least partially, so they can be separated by ion-exchange mechanisms. So another possible maltitol analysis method is high-performance anion-exchange chromatography (HPAEC), which is usually coupled with PAD, and allows the quantification of non-derivatised sugars and sugar alcohols at low concentrations in the order of pmol. This method is characterised by its high sensitivity, and it neither entails complex sample pretreatments nor uses organic solvents [23].

PAD can be interesting to determine maltitol as it directly detects it (with no pre- or post-column derivatisation) and offers high sensitivity and selectivity with complex commercial samples. PAD only detects analytes with functional groups that are oxidisable at the specific voltage applied to detection which makes the required sample preparation simple. Sodium hydroxide gradients can be used, as detection is not affected by salt concentration changes [23]. Cataldi et al. set up an HPAEC-PAD method for separating and quantifying four sugar alcohols and five sugars, including maltitol, in cakes, biscuits, toffees, creams, chocolate, chicory and roasted malt [35]. The extraction was performed with water under sonication, followed by centrifugation and filtration. A treatment with Carrez reagents to remove proteins and fats was applied in some products, followed by dilution and filtration (0.2 µm nylon membranes) prior to injection. A pellicular column with a relatively low ion-exchange capacity was successfully employed. It is well-known that data reproducibility in HPAEC is strongly affected by the interference of carbonate ions, which tend to occupy the column’s active sites, and thus reduces the retention of both sugar and sugar alcohol molecules. Regardless of taking all the precautions when preparing the alkaline eluent, this divalent ion still remains. The authors have shown that the presence of barium ions in the alkaline mobile phase increases both selectivity and reproducibility, but cuts analysis times, because it enables the precipitation of carbonate ions. This means that either taking precautions while preparing the alkaline eluent or regenerating the column between runs is no longer necessary. The method was applied to commercial samples. Maltitol was found in all the matrices, except for sponge cakes and creams. The levels detected in chicory and roasted malt were very low [35]. Andersen et al. also proposed a HPAEC-PAD method for quantifying six sugar alcohols and four sugars, including maltitol [26], but the kit performed poorly when separating maltitol and fructose [26].

Despite the merits of the different aforementioned detectors, mass spectrometry (MS) is still widely employed in both qualitative and quantitative analyses of sugars/sugar alcohols as it is able to identify compounds with high levels of sensitivity and specificity. MS allows maltitol to be directly detected without derivatisation. In this context, electrospray ionization (ESI) is normally coupled with HPLC-MS and MS/MS systems [19]. Shah et al. developed a method to simultaneously determine 14 non-nutritive sweeteners, including maltitol, in food and beverages by ESI with UHPLC (ultra-high-performance liquid chromatography) MS/MS in the negative ion mode [36]. The method required minimal sample preparation/clean-up. The beverages were simply diluted with water, except those containing gas, which were first sonicated to remove it. Hard candies were dissolved in water, vortexed and diluted. Yoghurts were processed using SPE. All samples were filtered (0.20 µm membrane filters) prior to injection. The authors tested different kinds of reversed-phase columns, including C8 and C30. The C18 column performed best, i.e., with easier and faster separation. The method enabled the quantification and monitoring of all the analytes by multiple reactions with three isotopically labelled internal standards. Obtaining two structurally significant MS product ions for both analytes and internal standards led to more selectivity and confirmation, which implies a major advantage [36].

Coupling MS with chromatography offers a powerful technique to selectively determine sugars and sugar alcohols in a single run. However, this detection method requires costly analytical instruments and specialised technicians [38]. High-purity organic solvents are also necessary for liquid chromatography [40]. An alternative analytical technique is capillary electrophoresis (CE), which offers good separation efficiency with low operating costs and minimal waste and has, therefore, been increasingly used to study several types of analytes in relatively complex matrices. The separation principles of sugar alcohols in CE systems are similar to those which apply to HPAEC [40].

The usability of CE in analysing maltitol has been recently proven by Coelho et al., who proposed using a method with CE and capacitively coupled contactless conductivity detection (C^4^D) [37]. The target analytes were four sugar alcohols, which included maltitol. Chocolate was the studied matrix. Extraction was performed using water and ultrasound, followed by filtration (0.22 µm membrane filters) and dilution. The strong alkaline background electrolyte was 25 mM of sodium borate (pH 8.5), which allowed negatively charged borate esters to form, produced by the borate ions-sugar alcohol interaction. The separation of all analytes was achieved in an impressive time: less than 6 min. This method was applied to commercial chocolate samples and revealed that most contained maltitol as the main sweetener [41].

By way of conclusion, the method of choice for maltitol determination in different food matrices is HPLC because it is compatible with its physico-chemical characteristics, it is able to meet multi-analyte detection needs, and demonstrates simplicity, high sensitivity and robustness. CE is an interesting alternative that often incurs lower running costs. However, it would appear that its robustness is limited [19]. Repeatability and reproducibility issues have been pointed out by several authors, which are mainly caused by inconsistent injection amount and unstable electroosmotic flow rate in the capillaries [42]. Some quantitative studies have directly compared the performance of CE and HPLC. In research conducted by Prado et al. CE provided a swifter analysis, whilst RP-HPLC displayed both superior repeatability (relative standard deviation (RSD) 0.98% vs. RSD 1.62%) and sensitivity (three times less LOD (limit of detection)) [43]. Similarly, Velikinac et al. reported higher selectivity for CE method, but the HPLC method was more sensitive (LOD 2.5 times less) and delivered superior precision (RSD ˂ 2% vs. RSD ˂ 4.5%) [44]. Gas chromatography is not a popular option given the need for derivatisation [34]. As UHPLC-MS/MS systems become increasingly common in laboratories, more methods using this technique will be developed and will enable foodstuffs to be analysed at high-throughput levels. Multi-analyte methods are most advantageous [19]. For the time being, the development of robust reliable methods to determine maltitol and other sweeteners in complex food matrices is an omnipresent challenge. Sound analytical methods are crucial for fulfilling growing needs in the food quality and safety fields.

## 3. Applications in the Food Industry and Safety

Huge efforts have been made to cut sugar intake in many food products for health reasons, particularly related to cardiovascular diseases and diabetes. This worrying phenomenon has attracted a ‘sugar tax’ that is imposed on food and drink industries in many countries. As confectioneries are more frequently consumed in the Western world, replacing sucrose is an ongoing challenge for food product developers to focus on alternatives that match aromas, sweetness, mouthfeel and texture. Maltitol forms part of the polyol group used for replacing sugar in the food industry. Polyols are generally relevant to the food industry for combatting weight control, diabetes and tooth decay.

Maltitol is a sugar alcohol that comes from maltose, a disaccharide with a similar sweetness to that of sucrose. Maltitol has many applications in food products like bakery and dairy products, chocolate and sweets. It is commercially available as maltitol syrup (E965ii) and crystalline maltitol (E965i) and is already included in a wide range of foods like chewing gum, chocolate, tableted mints, and other related products such as hard and chewy sweets.

Maltitol is a promising alternative to sugar as a bulk sweetener because its sweetness is almost 85–95% of that of sucrose. Its hygroscopicity is low, and it possesses excellent flow properties and a crystalline structure that results in end products with a very good taste and mouthfeel. Maltitol has a low glycaemic response of 29 and a calorie value of 2.4 kcal per gram in both Europe and the USA, which are lower figures than for traditional bulk sweeteners like sucrose [2]. The glycaemic index (GI) of crystalline maltitol is 36 and the GI for syrup is 52 with a higher hydrogenated oligosaccharides content [45]. Table 2 compares some unique properties of maltitol are compared to those of sucrose.

Given its many physico-chemical properties, the replacement of sugar with maltitol and its application in foods makes it extremely versatile. The main considerations for the food industries include cooling effect, solubility, hygroscopicity in response to relative humidity, sweetness and taste [46]. Maltitol can act as a bulking agent, humectant, emulsifier, sweetener stabilizer, or thickener in food and drink applications [41].

Awareness about eating low-calorie sweeteners, not only in patients with diabetes but also in the general population, has increased because sweeteners are employed as ingredients in many low-calorie foods: e.g., powdered drink mixes, soft drinks, dairy products, desserts, baked goods, chocolates, sweets, puddings, canned foods, jams and jellies, confectionery and chewing gum. Low-calorie sweeteners can be employed as table-top sweeteners at home, in restaurants and in cafeterias [47].

This section discusses how these properties offer advantages for developing food products, with further details in the following sections on metabolism and health impacts.

Maltitol’s sweetness is pleasant and clean and accounts for up to 90% of that attributed to sucrose, but its calorie value is 2.1–2.4 kcal/g (Table 2). Maltitol is also a non-cariogenic agent. Given its slow absorption, the insulin response associated with its ingestion significantly reduces. When applied simultaneously with short-chain fructo-oligosaccharides in sugar-free food product formulations, it lowers postprandial glycaemic responses [41]. As its hygroscopicity is low and it remains stable at high temperature, it is used in many baked products, and also as a variety of low-fat, reduced calorie and sugar-free food. Given its similarities to sucrose, maltitol can replace it in many formulations on a weight-for-weight basis. This allows many healthy snacks to be produced, including “sugar-free” or those with “no added sugar.”

A summary of common food products in which sucrose is replaced with maltitol is shown in Table 3.

Common maltitol applications include reduced-sugar baked goods, in which it can act as a 1:1 replacement for sucrose. Its sweetening and bulking properties are equivalent to those of sucrose. For instance, addition of maltitol significantly impacts the quality of dough and bread by affecting water mobility, thermal properties and retrogradation, which denote structure-function relations. Maltitol can lower dough fermentation rates and specific bread volumes to a certain extent. One study has shown that the presence of maltitol can rise the gelatinisation temperature of wheat starch and reduce its enthalpy.

Hardness and chewiness of bread tend to become lower when maltitol is applied, which suggests that maltitol can improve bread tasting properties. Low Field-Nuclear Magnetic Resonance data reveal a weaker interaction between water and starch chains or the gluten network when maltitol is present [1]. This study demonstrated that maltitol performed a binding potential with water and retarded the staling of bread. These phenomena are attributable to hydrophilicity and the hydroxyl number in maltitol, and to its further restriction in water mobility. The crust lightness of maltitol biscuits decreased by 25% because Maillard reactions did not occur, and biscuit texture was significantly softer with significantly better overall acceptance [1]. This study showed that maltitol can be a potential food additive to improve taste and to hinder the staling of bread. Nonetheless, more accurate comprehensive research into the interactions among maltitol, starch and the gluten network, and more rheological experiments, are needed.

Maltitol’s low hygroscopicity makes it a polyol of choice for chocolate applications because it contributes to high stability during conching and storage. Maltitol’s low hygroscopic character allows chocolate to be refined under the same conditions as sucrose and for conching at temperatures up to 80 °C. Addition of sweeteners to chocolate helps to cut cocoa bitterness, and its impact on rheological properties is also important for chocolate in end-product quality terms. Sucrose composition in chocolate is about 40–50%, depending on the type, which confers many functional properties to chocolate like sweetness, mouthfeel, texture, or particle size distribution (PSD) [48]. Employing maltitol as an alternative sweetener in chocolate improves its textural and sensory characteristics and enhances its storage stability due to anti-blooming effects [49].

The influence of maltitol and xylitol as bulking agents on the rheological properties of compound milk chocolate with a simplex-lattice mixture design has been investigated. The results showed that using maltitol and xylitol instead of sucrose can provide low-calorie compound milk chocolate with no undesirable rheological effects on samples. The results demonstrated that chocolate combinations containing 87.8% maltitol and 12.2% xylitol are optimum concentrations that produce the most acceptable rheological properties [50].

Drinkable yoghurts and flavoured milks have become increasingly popular in recent years as an alternative to high-sugar beverages, and also as delivery systems for prebiotics. High-potency sweeteners and hydrocolloid stabilisers are used to lower these products’ energy content. This approach gives a product with a distorted texture and an unpleasant mouthfeel. A more practical approach is to add maltitol or maltitol syrup to replace sugar solids because maltitol significantly contributes to these products’ overall sweetness and texture [51]. It is important to ensure that the polyol content of food products remains below 20 g per serving to avoid possible laxative effects. With maltitol, developed end products not only have a better texture because of more equivalent solids but also a more rounded sweetness profile.

Maltitol and maltitol syrups can be suitably applied to bakery products, chocolate and hard sweet production as they neither participate in Maillard reactions nor alter the product’s attractive appearance during thermal processing [51].

Maltitol is a useful fat substitute and sugar replacement in frozen dairy food and ice cream as it makes products creamy, sweet and sticky, and extends their shelf life. This makes the freezing point and sweetness of no-added-sugar ice cream similar to that of full-sugar ice cream for the same molecular weight, and sucrose results in a low-glycaemic-index ice cream without its texture being compromised. Taste and sweetness can, thus, be adjusted for sensory optimisation with a combination of sugars, including maltitol and sucralose supplementation to boost sweetness whenever necessary [52]. Dairy flavour and desire for sweetness strongly correlate with vanilla flavour perception that is lacking in alternative formulations. From the studied product formulations, a combination of tagatose (6%), polydextrose (6%) and maltitol (3%) or maltitol (15%) and trehalose (2.5%) in a formulation with milk cream and milk protein concentrate proves to be a potential formulation to meet both sensory and physico-chemical requirements [52].

Hard sweets are essentially constituted of sugar syrup (sucrose and glucose syrup in traditional products) that has been heated to reduce the moisture content to a very low level, insofar as the product’s glassy state remains upon cooling [53]. Aerated confectionery, such as marshmallow formulated with maltitol syrup and maltitol powder to replace glucose syrup and sucrose, has been successfully developed. Maltitol syrups containing 55–65% maltitol make good products, in which maltitol forms the major component of these formulations up to 70% of dry solids. For dusting powder applied to outer surfaces, a fine granulometry maltitol powder provides stable products, and its use is preferred to other polyols [53]. It can be used as a sweetener for sugar-free soft sweets and prevents other polyols in the formulation being obtained from crystallising. Sweets made with maltitol have better chewing and are not as sticky.

Maltitol syrup is an excellent polyol for pectin jellies. While producing pectin jellies, their final moisture content can slightly increase (0.3 ± 0.5%) to obtain a similar texture and shelf life to traditional glucose sucrose jellies, and a small amount of carrageenan (0.4 ± 0.5%) can be added to increase the firmness of products stored at high temperature and humidity [46]. These authors also recommended a higher maltitol content of almost 75% to help to prevent pectin from setting too quickly, and they advise depositing at temperatures above 90 °C [46].

In a recent study into reduced sugar jellies formulation from *Physalis peruviana* L. fruit, otherwise known as Cape gooseberry, Inca berry, Peruvian groundcherry, or goldenberry, the authors showed that the physicochemical and textural properties of jellies made their use promising in different foods with a sweet taste, and offer further opportunities for future research into product development [54]. These authors suggested that maltitol and maltitol syrup sweetener are the most functional sugar substitutes in the composition of jellies from physalis juice, as jelly with maltitol and maltitol syrup had a 90% lower total sugar content than that of the jelly made with sucrose and 83% of that produced with fructose.

In addition, the jelly obtained with maltitol and maltitol syrup had the lowest energy value compared to jellies obtained with sugar and fructose. Henceforth according to the terms of EU Regulation No. 1924/2006, jellies with physalis juice and maltitol/maltitol syrup can be classified with nutrition claims such as “food with no added sugars” and “energy-reduced food” [54].

Maltitol is generally recognised as ‘halal’ as it is permitted by Islamic law and it is kosher pareve as it meets all “kashruth” requirements. It is regarded as vegan-friendly and is GMO-free because raw material starch comes from non-GMO plants [45]. Depending on the overall formulation, products containing maltitol can display a number of label claims, including “no sugar added”, “sugar-free” and “low-calorie”.

According to the evaluation of the European Food Safety Authority (EFSA) and the Codex Alimentarius Commission, maltitol and other low-calorie sweeteners are safe for human consumption and will not cause cancer or other health-related problems as long as they are consumed at acceptable daily intakes (ADI) [47]. Similarly, extensive toxicological testing by the Joint FAO/WHO Expert Committee of Food Additives (JECFA) has shown that maltitol syrups are safe for human consumption and were given an ADI status. The EFSA is currently reviewing the technico-toxicological data on maltitol and other sweeteners that are authorised as food additives in the EU. This on-going re-evaluation is expected to be completed by the end of 2020 [55].

As maltitol is fermented in the colon, its digestion rate is slow and it is not completely digested, which means a slower rise in blood sugar and insulin levels compared to glucose and sucrose [2] in a study that evaluated the impact of confectionary sweeteners on the composition of gut microbiota. An optimal dose of 34.2 g for maltitol plus polydextrose significantly increased the numbers of faecal bifidobacteria, lactobacilli, and short-chain fatty acids after maltitol ingestion compared to sucrose intake [17]. To date, however, not enough data are available to determine the specific effects of maltitol on gut microbiota, so more studies are necessary [47].

Compared to the reactions that occur after consuming standard sucrose-containing chocolate, the occasional or regular consumption of increasing doses of maltitol is not associated with significant digestive symptoms but does result in increased diarrhoea [10]. Generally speaking, most polyols, including maltitol, have a few side effects when overeaten, such as laxative, gastrointestinal symptoms, bloating, diarrhoea and abdominal pain. Therefore, if any food product contains more than 10% added maltitol or other polyols, it must include the statement “excessive consumption may have laxative effects” [56].

## 4. Metabolism

Maltitol has substituted for sucrose in many food applications on a weight-for-weight basis due to its slow unfinished absorption through the small intestine and fermentative degradation by intestinal microbiota [4]. The laxative effect of polyols is frequently cited as the main cause of their lack of market penetration (other than their cost vs. sugars). Luckily however, maltitol is one of the best tolerated polyols.

The main features of the metabolism of polyols xylitol, sorbitol, erythritol, mannitol, isomalt, lactitol, maltitol and hydrogenated starch hydrolysates have been described by many authors [57,58,59]. They include their metabolism, absorption and glycaemic effects, energy utilisation, gastrointestinal tolerance and dental properties. Although structural similarities exist between these polyols, data reveal that the metabolic characteristics of each polyol, especially their calorific values, have to be individually considered [4]. Moreover, we should consider each person’s specific condition. Basically, studies on maltitol metabolism have focused on its degradation in the intestines and its effects on the adaptation of dental plaque to metabolise maltitol. Thus, we focus on these two topics.

Maltitol is added to many foods as a sweetener and sugar substitute in relation to its possible interaction with different ingredients. Some studies have reported that the concentration of a sweetener and the characteristics of foodstuff remain stable throughout their shelf life and do not significantly vary when looking at normal food products: yoghurt, burfi, flavoured milk [29], and chocolate [49].

Regarding the oral metabolism of maltitol, it is noteworthy that the oral cavity is a complex ecosystem with the most diverse microbial populations and the second largest diverse microbiota after the gut, with over 700 species of bacteria [60]. In the oral cavity, most habitats are dominated by *Streptococcus*, and other species are located in different areas, followed by *Haemophilus* in buccal mucosa, *Actinomyces* in supragingival plaque, and *Prevotella* in the immediately adjacent (but low-oxygen) subgingival plaque [61,62].

A very important element of pH regulation is saliva (pH 6.5–7), with buffer capacity in the oral cavity. Thus, saliva pH lowers more after consuming snacks containing maltitol. After 10, 15, 20 and 30 min, significant changes take place in saliva pH compared to the initial pH (the zero minute) [63]. Some research has found [63,64] that patients at high caries risk have a significantly lower saliva pH than patients at low caries risk. The type of sweetener in snacks also affects saliva pH, as shown by a reduction after patients have eaten snacks containing sucrose compared to those who ate snacks containing maltitol. This may be because *Streptococcus mutans* cannot change maltitol into acid because essential enzymes are lacking, even though maltitol can penetrate into the membrane of the bacteria cell, which reduces the activity of glucosyltransferase [65] Sucrose can easily be fermented into lactic acid and piruvic acid and, thus, enhance the enzymatic activity of glucosyltransferase [66].

For other polyols, it has been demonstrated both in vivo and in vitro that xylitol and sorbitol inhibit the growth of a number of cariogenic bacteria, including *Streptococcus mutans* and *Streptococcus sobrinus* [67,68,69,70,71]. The mechanism is assumed to be due to the accumulation of sugar alcohol in the cell upon uptake, which results in the formation of a toxic sugar phosphate [71,72]. The effect of maltitol on dental plaque has been studied by Keijser et al. [57], and knowledge about its microbiota is a key element in studying the degradation of polyols. Upper buccal plaque microbiota is dominated by members of the phylum Proteobacteria (30.9%), Firmicutes (26.4%), Actinobacteria, (19.2%) Bacteroidetes (14.0%) and Fusobacteria (9.0%). Lower-lingual plaque is dominated by Fusobacteria (28.2%), Bacteroides (20.6%), Proteobacteria (19.8%), Firmicutes (18%) and Actinobacteria (12.0%). The dominant genera in upper-buccal plaque samples are *Haemophilus*, *Streptococcus*, *Corynebacterium* and *Neisseria*. Lower-lingual plaque samples are dominated by *Leptotrichia*, *Fusobacterium*, *Prevotella*, *Veillonella* and *Capnocytophaga*. The effects of polyol-sweetened gums on healthy oral microbiota have not yet been established [57].

Another study indicates that dental plaque adjustment in order to metabolise sucrose and sorbitol occurs with frequent exposure to these sweeteners, while frequent exposure to maltitol and xylitol does not result in plaque adjustment to metabolise these sweeteners [65].

Regarding the intestinal metabolism of maltitol, it is relevant to consider that low-digestible carbohydrates are consumed incompletely, or not, in the small intestine, but are at least partially fermented throughout the large intestine by bacteria [73]. Maltitol belongs to this group (glucose plus sorbitol) as it is included in polyols. Carbohydrates of at least two units usually need to be reduced enzymatically into monosaccharides before they can be absorbed in the small intestine and enter circulation.

During a clinical trial, the consumption of maltitol-polydextrose chocolate contributed to intestinal prebiotic effects, but these findings cannot be clearly extrapolated due to the mixture of putative functional ingredients in the experimental diet [17]. Tolerance to slowly absorbed bulking sweeteners like sugar alcohols is often assessed by fasting healthy volunteers eating large or increasing quantities of the test substance, either on its own or diluted in water [10]. Stool excretion after ingesting sugar alcohols is negligible, which implies that sugar alcohols reach the large intestine when they are almost completely digested by colonic flora [74]. Yet this malabsorption has certain side effects, such as the fermentation of unabsorbed sugar leading to flatulence. As polyol molecules are osmotically active, diarrhoea may occur when the ability of colonic flora to ferment these low-molecular-weight carbohydrates is surpassed and osmotic stress arises in the intestinal lumen [75]. Regular maltitol consumption did not lead to increased rectal gases and in breath H_2_ production did not lower compared to occasional maltitol consumption. This means that regular maltitol consumption did not result in colonic flora adaptation and was variable with different unabsorbable sugars [76]. These results demonstrate that a larger part of ingested maltitol is fermented by intestinal microbes than is hydrolysed by digestive enzymes. Although maltitol is catabolised to carbon dioxide via intestinal microbes, there is much less available energy than that of digestible sugars like sucrose [8].

Several carbohydrates, such as isomalt, sorbitol and lactitol, were worse tolerated than similar amounts of maltitol [9]. We only found one study that has compared the effects of maltitol to those of polyglycitol [74], but with different amounts of both. It can y be inferred that certain sugar alcohols are just as well tolerated at the respective intakes (no diarrhoea or other side effects).

According to research into rats and humans, Hosoya [77] concluded that maltitol would not be hydrolysed, but only slightly absorbed from the small intestine before being quickly excreted again. Other investigators, however, stated different degrees of absorption. The detailed study of Rennhard and Bianchine [69] concluded that maltitol is partially hydrolysed in the stomach, partially absorbed intact from the small intestine, and partially degraded by gut flora to volatile fatty acids, which are easily absorbed and utilised by the microbiota.

According to research into rats and humans, Hosoya [77] concluded that maltitol would not be hydrolysed, but absorbed only slightly from the small intestine before being quickly excreted again. Other investigators, however, stated different degrees of absorption. The detailed study of Rennhard and Bianchine [78] concluded that maltitol is partially hydrolysed in the stomach, partially absorbed intact from the small intestine, and partially degraded by gut flora into volatile fatty acids, which are easily absorbed and utilised by the microbiota.

Another benefit of several sweeteners is the relation with H_2_ production in the small intestine, which can reduce hepatic oxidative stress, diminish the severity of neurological disorders [79,80], lead to lower concentrations of inflammatory cytokines [81], and has been employed as a drug for the suppression of postprandial hyperglycaemia by inhibiting the digestion of disaccharides. It can also suppress the risk of myocardial infarction in patients with type 2 diabetes [82].

Hydrogen breath studies have indirectly tested malabsorption by measuring hydrogen expiration, a result of colonic fermentation. The major source of exogenous H_2_ is the intestinal microbiome which produces it from indigestible components whose source can be dietary fibre (non-digestible carbohydrates and lignin), including functional fibre (isolated non-digestible carbohydrates shown to have beneficial physiologic effects on humans) [83], and can be responsible for inducing H_2_ production. Matsumoto et al. [84] concluded that a combination of the different chemical structures of indigestible components, such as H_2_-producing milk containing sugar alcohol (maltitol), may be important and effective for H_2_ production by various intestinal microbiomes (Rikenellaceae, Clostridiales Incertae, Clostridiales, Ruminococcaceae and *Alistipes*).

Table 4 summarises several benefits of the regular consumption of maltitol versus other sweeteners.

## 5. Impacts on Health

Thorough toxicological studies have proven that maltitol is safe for use. The Joint FAO/WHO Expert Committee of Food Additives (JECFA) has assigned it as an acceptable “not specified” daily intake [85,86,87]. It is authorised in most countries for food use, although some countries (EU, USA) have their own particular legal standards and purity specifications for this substance, while others do not and instead follow the Codex Alimentarius specification for maltitol. Polyols are known as food additives in the EU, and their use in food is regulated by Sweeteners in Food Regulations. Maltitol (and maltitol syrups) have been assigned E number E965 and are approved for use in food at quantum satis for a variety of food items specified in the regulations, usually bakery goods, confectionery, ice cream, desserts and fruit preparations. Polyols are not usually permitted in drinks (except erythritol) due to over-consumption laxation issues. Maltitol (and other polyols) cannot be used in foods in association with sugars in the EU unless the polyol is employed, as a result of the mixture, for a technical purpose other than sweetness or towards a 30% reduction in calories that results from the combination. Prohibitions on using polyols in conjunction with sugars do not exist in the USA, and the Food and Drug Administration (FDA) finds polyols to be a food additive or ‘Generally Recognized as Safe’ (GRAS). Maltitol has a self-affirmed GRAS status [4,88]. Of all sugar alcohols, maltitol most resembles sugar flavour. It is not cariogenic and is safe for diabetics [89,90].

Its usage does not facilitate tooth decay [6,88,91] because it cannot be fermented by oral bacteria [7,92]. Daily use of gum containing maltitol induces the inhibition of some bacteria present in supragingival plaque microbiota, many of which are known as first dental surface colonisers [57]. Several in vitro and animal experiments [93,94] have identified the oral health impact of maltitol, as have clinical trials [95,96]. Yet maltitol’s health advantage is not restricted to dental care. Evidence from previous research works indicates that maltitol can have major anti-hyperglycaemic potential [3,6,97,98]. This is because a single oral administration of 50 g of maltitol to healthy individuals resulted in substantially lower glycaemic and insulin responses compared to administering the same volume of glucose or sucrose [99,100]. It has also been documented that the intake of 30 or 50 g of maltitol as a single oral dose leads to a lower glucose and insulin response compared to diabetic subjects’ ingestion of an equal volume of maltose or glucose [101,102]. Overweight participants fed low-fat, low-calorie and high-amylose cornstarch-sweetened maltitol muffins displayed lower glucose, insulin and lipidaemic responses, but improved satiety versus those fed traditional sugar-sweetened muffins [98]. Two prior reports recorded a single oral maltitol administration, namely sorbitol mixture (60:7) [100] or 50 g of maltitol [103], which significantly reduced blood insulin and glucose responses in relation to administering the same volume of glucose in both normal and diabetic participants.

A study by Kang et al. [104] documented in vitro alpha glucosidase, alpha amylase and sucrase inhibitory maltitol activity, which indicates that maltitol can be useful for regulating carbohydrate digestion and postprandial hyper-glycaemia. Contrarily to this, Matsuo [97] stated that maltitol did not inhibit intestinal glucosidase, sucrase or maltase operations with a single oral dose of a maltitol and sucrose mixture (25:25 g) or of sucrose (50 g) or maltitol (50 g) given to healthy individuals. Based on the findings of the above-mentioned studies, it can be argued that maltitol may show a hypoglycaemic reaction by other mechanisms, such as inhibiting the absorption of intestinal glucose and/or increasing muscle glucose uptake, rather than inhibiting intestinal carbohydrate digesting enzyme activities [105]. Thabuis et al. [3] also noted that, in accordance with FAO recommendations, the maltitol glycaemic response (GR) was significantly lower than the glucose GR up to 90 min after its administration. The insulin-emic response (IR) to maltitol was substantially lower than the glucose IR up to 2 h after administration, according to FAO recommendations. Maltitol showed few glycaemic and insulinaemic responses. Both substances were well tolerated by all the volunteers who participated in the study up to a single intake of 50 g.

Chukwuma et al. [105] observed that maltitol prevents the synthesis of intestinal glucose and improves the accumulation of insulin-mediated muscle glucose ex vivo, but not in normal and type 2 diabetic rats when co-ingested with glucose. Dietary glucose is consumed quickly from the small intestine. However, experiments in vitro have indicated that the proximal [106] or mid-small intestine (part of the duodenum and jejunum) [107] shows the highest glucose absorption. Hence the jejunal rat intestine portion was used in the ex vivo procedure to study glucose absorption. It is also recognised that osmotic pressure can affect the absorption of intestinal water and glucose. Previous in vitro research has indicated that lowering osmolality in isolated rat duodenums enhanced the uptake of luminal water and glucose [108].

The peak value of 5.20 ± 0.72 mg/cm jejunum of glucose absorption was found in the ex vivo absorption research by Chukwuma et al. [105], which lowered to 3.00 ± 0.35 mg/cm jejunum in the presence of 2.5% maltitol. Maltitol’s inhibitory effect on jejunal glucose absorption was concentration-dependent, with the lowest value of 1.20 ± 0.20 mg/cm jejunum at 20% maltitol. These results indicate a concentration-dependent inhibitory influence of maltitol on ex vivo jejunal glucose absorption, which may be attributed partly to a growing osmolarity (388.48–896.68 mOs m/L) influence of increasing maltitol concentrations (2.5–10%). Unlike the major ex vivo inhibitory effect of maltitol on jejunal glucose absorption, however, a single oral maltitol administration that was co-ingested with glucose did not significantly affect or lower the glucose absorption index in intestinal segments. This effect similarly translates into the observed marginal effect in normal glycaemic and diabetic animals on postprandial blood glucose levels. As seen in several previous studies [78,109,110], this finding under in vivo conditions may be related to maltitol hydrolysis by disaccharidases of small intestinal mucosa. The recent findings of Chukwuma et al. [105] tend to further clarify the apparent inconsistency between the documented effects of maltitol on alpha glucosidase and alpha amylase, which ranged from in vitro [104] to in vivo experimental conditions [97].

Gastric emptying and digesta gastrointestinal tract transit levels primarily affect nutrient absorption in the small intestine. Previous research has shown that delayed gastric emptying and rapid digestive transit can lead to less intestinal absorption of nutrients and food intake [111,112], which is believed to be a mode of action of acarbose in regulating postprandial blood glucose production in diabetic patients [113]. A single oral dose of maltitol has recently been shown to have no major impact on gastric emptying in normal or diabetic animals, which could be partly responsible for the negligible effect of maltitol on small intestinal glucose absorption [105]. Nevertheless, maltitol accelerated the digestive transit in the caecum of diabetic rats, but not other parts, which did not affect overall intestinal glucose absorption because most glucose absorption occurs in the first quarter to the mid-small intestine [106,107].

Circulating glucose uptake by cells is a significant mechanism for the body to preserve glucose homeostasis, as blood glucose rises owing to glycogen degradation, dietary glucose absorption and gluconeogenesis [114] for either storage or energy metabolism. Insulin is the main controlling hormone in activating the clearance of circulating glucose through insulin-mediated glucose absorption in cells [114]. Some previous studies have stated that hyperosmolarity improves muscle glucose absorption by AMP-Kinase (adenosine monophosphate-Kinase) regulation and/or glucose transporter type 4 endocytosis inhibition [115], but recent ex vivo research has revealed that while maltitol demonstrates insulin-mediated glucose uptake (GU50 = 7.31 ± 2.08%), it does not have any substantial insulin-free glucose uptake effect on isolated rat psoas muscle (GU50 = 111.12 ± 19.36%) [105]. This result may indicate that maltitol can potentiate insulin-mediated glucose absorption in muscles, at least in ex vivo environments, when increased osmolarity (388.48–896.68 mOsm/L) due to higher maltitol concentrations cannot be an influential factor in this regard.

Regarding maltitol’s possible genotoxicity and teratogenicity, a few decades ago Takizawa and Hachiya [116] stated that maltitol was not mutagenic in *Escherichia coli* and *Salmonella typhimurium* strains and did not induce the frequency of micronucleus in mice bone marrow cells. Canimoglu and Rencuzogullari [117] also stated that maltitol did not induce the mean of sister chromatid exchanges and the percentage of chromosome aberrations at all concentrations and for all treatment periods in human lymphocytes but did induce the micronucleus frequency with no dose dependency.

In a study by Canimoglu and Rencuzogullari [118], conducted to reveal the genotoxicity and cytotoxicity of maltitol, rats were intraperitoneally administered with up to 10 g/kg body weight (bw) maltitol concentrations; that is, a very high dose. Despite this dose, maltitol was neither genotoxic nor cytotoxic. It ought not to be believed that 10 g/kg bw of maltitol should be given to humans in one day. Hence the maximal employed maltitol dose cannot be surpassed, and it can be inferred that maltitol has no genotoxic impact on the in vivo test system.

Maltitol was not proven cytotoxic in rat bone marrow cells because it did not lower the mitotic index. Maltitol was not cytotoxic in human lymphocytes [117], but for other sweeteners there are reports that some were cytotoxic [119,120,121] and some were not [122]. Maltitol was not shown to be teratogenic, but an embryotoxic influence was demonstrated by reducing the weight of foetuses and inducing growth retardation at a very high concentration (4 g/kg bw) [112].

Therefore, at lower doses, it can be inferred that maltitol poses a low risk for humans but may induce diarrhoea when taken at high doses [112]. Additionally, maltitol may induce hyper-glycaemia and lower embryo weight during pregnancy when used at high doses over long periods, particularly during the first trimester of pregnancy [112]. Accordingly, caution must be taken in utilising it at higher concentrations in food and beverages for the health of our generation and future generations. Table 5 summarises the main attributes of consumed maltitol and its relations to health impacts.

## 6. Conclusions

Maltitol remains innocuous and helps to improve consumers’ health quality due to benefits such as exerting prebiotic effects, lowering calorie consumption due to sucrose, and promoting dental health. Due to the similarity of the physicochemical features of maltitol and sucrose, the latter can be easily substituted for maltitol in several foodstuffs; we therefore considered several analytical methods in the determination of maltitol in food samples and its identification can be executed by HPLC methods, which are the most widely used analytical methods of choice. When considering the pros and cons of different analytical methods, HPLC is easier and widely used to detect maltitol in foods.

Based on our literature review, in order to gain a better understanding of the metabolism of maltitol and its impacts on human health, more studies need to be conducted to determine the effects of larger maltitol doses over longer periods of time on gastrointestinal tolerance, gut microbiota in both the small and large intestines, and oral cavity. Since a high consumption of maltitol has some adverse effects such as flatulence and laxative effects, adequate information has to be provided to consumers. Labelling foodstuffs containing sweeteners (>10%) that “excessive consumption may have laxative effects” is an important way to provide information on such effects.

## Figures and Tables

**Table 1:** Analytical procedures used in the determination of maltitol in food samples.

Analyte	Matrix	Technique	Sample Preparation	Mobile Phase/ Electrolyte	Column/ Capillary	Analytical Parameters	Ref.
**Xylitol** ***Meso*-erythritol** **D-glucitol** **D-mannitol** **Maltitol** **Parachinit**	Confectionery products	HPLC-UV (260 nm)	Extraction with 30% ethanol, followed by centrifugation, evaporation, and derivatisation with a 10% solution of *p*-nitro-benzoyl chloride. Excess reagent was destroyed and the sample was evaporated. The residue was dissolved in chloroform and purified in an SPE cartridge.	Acetonitrile–water (67:33)	GL Sciences stainless-steel column (250 × 4.5 mm) packed with Inertsil Ph-3	LOD 0.1% LOQ n.a. Recovery 73.2–109.0% RSD ≤ 9.0%	[27]
**Maltitol**	Milk Burfi Yoghurts	HPLC-RI	Extraction with water under sonication for 20 min at 40 °C. Treatment with Carrez reagents to remove proteins was applied, followed by filtration (filter paper and 0.22 µm syringe filter).	Acetonitrile–water (75:25)	Waters Spherisorb Amino column (5 µm, 250 × 4.6 mm) with a guard column Waters µBondpack (10 µm NH_2_)	LOD 10 µg/mL LOQ 25 µg/mL Recovery 97.81–98.54% RSD ≤ 1.93%	[29]
**Lactose** **Sucrose** **Fructose** **Glucose** **Xylitol** **Isomalt** **Sorbitol** **Erythritol** **Maltitol**	Desserts	HPLC-RI	Extraction with distilled water (50 °C) in a water bath 60 °C for 15 min, precipitation with sodium hydroxide and zinc sulphate, and filtration (0.45 µm membrane filters).	Distilled water	Shodex Sugars SP0810 column (300 × 8.0 mm) with lead (II) ions and a guard column Shodex SP-G (5 μm, 50 × 6 mm)	LOD 0.01–0.17 mg/mL LOQ 0.03–0.56 mg/mL Recovery 91–109% RSD ≤ 8%	[32]
**Fructose** **Sucrose** **Glucose** **Lactose** **Maltose** **Erythritol** **Sorbitol** **Xylitol** **Inositol** **Mannitol** **Lactitol** **Isomalt** **Maltitol**	Sweets Jellies Gums Chocola1te Processed chocolate products Snacks	UPLC-ELSD	Extraction with water at 80 °C for 30 min (gums and sweets) or 50% alcohols at 80 °C for 30 min after fat removal (chocolate and processed chocolate products), centrifugation, and filtration (0.22 µm PVDF syringe filter).	Acetonitrile (eluent A) and water (eluent B) both containing 0.05% (*v*/*v*) ethanolamine and triethylamine as modifiers	Acquity BEH Amide column (1.7 μm, 150 × 2.1 mm)	LOD 0.006–0.018% LOQ 0.020–0.059% Recovery 89.13–105.32% RSD ≤ 1.55%	[33]
**Maltose** **Sucrose** **Fructose** **Glucose** **Xylitol** **Sorbitol** **Erythritol** **Mannitol** **Maltitol**	Fruit juices Fruit beverages Nectars Dietary supplements (syrups)	HPLC-CAD	Filtration (through filter paper to remove solid particles), dilution with 75% acetonitrile, and second filtration (0.45 µm membrane filters).	Water (eluent A) and acetonitrile (eluent B)	Shodex Asahipak NH2P-50 4E column (5 μm, 250 × 4.6 mm)	LOD 0.12–0.44 μg/mL LOQ 0.40–1.47 μg/mL Recovery 95.6–105% RSD ≤ 4.97%	[25]
**Cl^−^** **K^+^** **Br^−^** **SO_4_^2−^** **NO_3_^−^** **Erythrose** **Arabinose** **Fructose** **Galactose** **Glucose** **Lactose** **Isomaltulose** **Maltose** **Lyxose** **Maltotriose** **Mannose** **Rhamnose** **Raffinose** **Ribose** **Sucrose** **Xylose** **Sorbose** **Erythritol** **Inositol** **Lactitol** **Mannitol** **Maltitol** **Xylitol** **Sorbitol** **Acarbose**	Energy drinks Beer Soft drinks Wine Coffee Milk Smoothies Tea Fruit juices Ketchup Yoghurts Honey	HILIC-CAD	Dilution in 60% acetonitrile and centrifugation. Samples with gas were degassed in an ultrasonic bath prior to dilution.	85% acetonitrile (eluent A) and 60% acetonitrile (eluent B), both with 10 mM of ammonium acetate adjusted to pH 8.25 with ammonium hydroxide	WATERS Acquity UPLC BEH Amide column (1.7 µm, 150 × 2.1 mm) and an Acquity UPLC BEH Amide VanGuard precolumn	LOD 0.032–2.675 mg/L LOQ 0.107–8.918 mg/L Recovery n.a. RSD ≤ 4.94%	[34]
**Glucose** **Xylose** **Fructose** **Sucrose** **Lactose** **Sorbitol** **Lactitol** **Isomaltitol** **Maltitol**	Biscuits Cakes Creams Toffees Chocolate Roasted malt Chicory	HPAEC-PAD	Extraction with water under sonication, centrifuged and filtered. Treatment with Carrez reagents to remove proteins and fats was applied to some products, followed by dilution and filtration (0.2 µm nylon membranes).	40 mM of sodium hydroxide + 1 mM of barium acetate	Dionex CarboPac PA100 column (250 × 4 mm) and a guard column CarboPac PA100 column (50 × 4 mm) A gold working electrode and a silver/silver chloride reference electrode were employed. The optimal detection potential was +0.10 V.	LOD 10–20 pmol LOQ n.a. Recovery n.a. RSD ≤ 2%	[35]
**Glucose** **Lactose** **Sucrose** **Maltose** **Xylitol** **Sorbitol** **Mannitol** **Lactitol** **Isomaltitol** **Maltitol**	Desserts Cakes Sweets Liquorice Wine Gums Chocolate Pastilles	HPAEC-PAD	Extraction with water (60 °C) for 4 h at room temperature, centrifugation, filtration (through a folded filter S&S, 592.5, diameter = 125 mm), dilution and second filtration (0.2 µm Minisart).	100% 1M of NaoH (eluent A) and 100% water (eluent B)	Dionex CarboPac MA1 column (250 × 4 mm) and a guard column Dionex CarboPac MA1 (50 × 4 mm)	LOD 0.3–1.1 mg/l LOQ 1–4 mg/L Recovery 85.8–107% RSD ≤ 5.2%	[26]
**Cyclamate** **Saccharin** **Sucralose** **Dulcin** **Aspartame** **Neoheperidine** **Dihydrochalcone** **Acesulfame** **potassium** **Alitame** **Neotame** **Rebaudioside A** **Stevioside** **Erythritol** **Xylitol** **Maltitol**	Carbonated and non-carbonated beverages Hard sweets Yoghurts	UHPLC-MS/MS	Beverages were simply diluted with water, except those containing gas, which were first sonicated to remove it. Hard sweets were dissolved in water, vortexed and diluted. Yoghurts were processed using solid phase extraction (SPE). All samples were filtered (0.20 µm membrane filters) prior to injection.	10 mM of ammonium acetate in water/methanol (98/2, *v*/*v*) (eluent A) and 10 mM of ammonium acetate in water/methanol (1/99, *v*/*v*) (eluent B)	Waters Acquity UPLC BEH C18 column (1.7 µm, 100 × 2.1 mm) with a Vanguard pre-column (1.7 µm, 5 × 2.1 mm)	LOD 0.1–1.8 ng/mL (drinks) and 0.1–2.5 ng/g (sweets and yoghurts) LOQ n.a. Recovery 70–114% RSD ≤ 15%	[36]
**Erythritol** **Xylitol** **Sorbitol** **Maltitol**	Chocolate	CE-C^4^D	Extraction with water and ultrasound, followed by filtration (0.22 µm membrane filters) and dilution.	25 mM of sodium borate, pH adjusted to 8.5 with boric acid	Fused silica capillary column (70 cm × 50 µm) C^4^D parameters were 2 V (peak to peak), 628 kHz	LOD 2.7–4.8 µg/g LOQ 9–15.9 µg/g Recovery 70–116% RSD ≤ 19%	[37]

LOD—Limit of detection; LOQ—Limit of quantification; RSD—Relative standard deviation; n.a.—Not available.

**Table 2:** A comparison of some physico-chemical properties for sucrose and maltitol.

Physico-Chemical Properties	Sucrose	Maltitol
Molecular weight	342	344
Sweetness	1.0	0.9
Solubility at 22 °C	67%	65%
Melting point (°C)	168–170	144–152
Heat of solution (cal/g)	4.3	−5.5
* ERH for water uptake (20 °C)	84%	89%
Calories (kcal/g)	4.0	2.4 (EU)
Glycaemic index (GI)	68	35
Molecular formula	C_12_H_22_O_11_	C_12_H_24_O_11_
Chemical structure	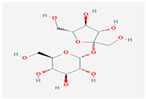	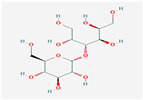

* ERH—Equilibrium relative humidity. Adapted from Grembecka [41].

**Table 3:** A summary of common food products where sucrose is replaced with maltitol.

Food Product	Impact of the Replacement on Quality Attributes
Reduced sugar baked goods	improved taste and reduced staling
Chocolate powder	improved textural and sensory properties
Milk powder	improved rheological properties
Frozen dairy foods and ice cream	improved creaminess, lower glycaemic index
Drinkable yoghurts and flavoured milk	reduced calorific content, better texture and sweetness profile
Candies and hard sweets	visual appearance is maintained during thermal processing
Pectin jellies	lower energy value, better physicochemical properties
Marshmallow	fine granulometry maltitol powder and increased stability

**Table 4:** Several benefits of the regular consumption maltitol versus other sweeteners.

Maltitol Consumption Benefits	Reference(s)
Does not reduce the saliva pH	[65]
Excellent relation with different ingredient of foodstuff	[29]
It is not fermented in the oral cavity	[66]
The oral cave micro-organisms cannot produce an adjustment to metabolise the maltitol	[65]
Possible probiotic effects	[17]
Produces less energy in small intestine	[17]
Partially hydrolysed in the stomach	[78]
Partially absorbed in the small intestine	[78]
Easily absorbed and utilised by the microbiota	[78]
Improvement of the H_2_ production by intestinal microbiomes	[84]

**Table 5:** The main attributes of maltitol for its consumption, taking into account impacts on health.

Attribute	Reference(s)
Not cariogenic	[89,90]
Prevents tooth decay	[6,88,91,95,96]
Antihyperglycaemic	[3,6,97,98,104,105]
Insulin-mediated glucose uptake	[105]
Not genotoxic	[118]
Not cytotoxic	[118]
Not teratogenic	[118]
Decreases foetus weight	[118]
Causes growth retardation at high doses (4 g/kg body weight)	[118]
